# Exploring the Link Between Metabolic Syndrome and Cellulite

**DOI:** 10.7759/cureus.63464

**Published:** 2024-06-29

**Authors:** Nikos Adamidis, Petros Papalexis, Sotirios Adamidis

**Affiliations:** 1 Internal Medicine, Sismanogleio Hospital, Athens, GRC; 2 Endocrinology, Laiko General Hospital, National and Kapodistrian University of Athens, Athens, GRC; 3 Internal Medicine, Athens Medical Group, Athens, GRC

**Keywords:** adipose tissue dysfunction, hormonal impalance, inflammation, cellulite, metabolic syndrome

## Abstract

Metabolic syndrome (MetS) encompasses a cluster of metabolic abnormalities, including insulin resistance, hypertension, abdominal obesity, and dyslipidemia, increasing cardiovascular disease and type 2 diabetes risks. Cellulite, a cosmetic condition marked by dimpled skin, predominantly affects women and shares risk factors with MetS, such as obesity and hormonal imbalances. This review examines the potential link between MetS and cellulite, focusing on shared pathophysiological pathways and implications for clinical practice and future research. Common factors such as inflammation, hormonal changes, and adipose tissue dysfunction are explored. The review highlights the importance of longitudinal studies to track cellulite progression in MetS patients, biomarker identification for early detection, intervention trials to assess therapeutic efficacy, mechanistic studies to elucidate underlying pathways and the impact of comorbidities on cellulite development. Understanding these relationships can enhance prevention, diagnosis, and treatment strategies for both MetS and cellulite, addressing significant public health and cosmetic concerns.

## Introduction and background

Metabolic syndrome (MetS) is a collection of interconnected metabolic disorders that greatly enhance the likelihood of developing cardiovascular illnesses and type 2 diabetes mellitus [1]. It is characterized by several important components, namely insulin resistance, hypertension, abdominal obesity, and dyslipidemia [1]. Cellulite is a prevalent aesthetic issue defined by a textured, uneven appearance of the skin, mainly impacting the thighs, buttocks, and belly [2].

The National Cholesterol Education Program Adult Treatment Panel III (NCEP ATP III) defines MetS as meeting at least three of the following criteria: having a waist circumference greater than 102 cm in men or 88 cm in women, having triglyceride levels of 150 mg/dL or higher, having high-density lipoprotein (HDL) cholesterol levels less than 40 mg/dL in men or 50 mg/dL in women, having a blood pressure of 130/85 mmHg or higher, and having fasting glucose levels of 100 mg/dL or higher [1]. Metabolic syndrome is a major public health issue because of its widespread occurrence and its link to higher rates of illness and death from cardiovascular diseases and diabetes [3].

The higher prevalence in women compared to men can be attributed to variations in fat distribution, connective tissue structure, and hormonal variables. Cellulite is the result of fat deposits beneath the skin pushing through the connective tissue, causing an uneven surface and a characteristic look resembling an 'orange peel' or 'cottage cheese' [2]. Although cellulite is not a medical condition and poses no significant health risks, it is a significant aesthetic concern for many individuals, affecting their self-esteem and body image [4].

Understanding the potential link between MetS and cellulite is critical, given the increasing prevalence of metabolic disorders and the high cosmetic concern associated with cellulite. Similar factors such as obesity, hormonal changes, and lifestyle choices influence both conditions and exploring their relationship could provide insights into potential common mechanisms and interventions. This review aims to examine the current evidence on the association between MetS and cellulite, highlight potential shared pathophysiological pathways, and discuss the implications for clinical practice and future research.

## Review

Common risk factors for MetS and cellulite

Age

Age is a significant risk factor for MetS, a cluster of conditions that increase the risk of heart disease, stroke, and diabetes. The prevalence of MetS increases with age, particularly after 40. Various factors, including age-related changes in metabolism, hormonal shifts, and an increase in sedentary behavior, are responsible for this rise [5]. As people age, cellulite, characterized by a dimpled appearance of the skin, becomes more common. This condition is more prevalent in women and tends to worsen with age due to changes in skin structure, reduced collagen production, and the accumulation of fat cells. As the skin loses elasticity and connective tissue weakens, the appearance of cellulite becomes more pronounced [3].

Gender

Gender plays a crucial role in the development of MetS, with men and women displaying different risk profiles. Men are generally at higher risk of developing MetS at a younger age. However, post-menopausal women are at an increased risk due to hormonal changes, particularly the decline in estrogen levels, which can lead to increased abdominal fat and insulin resistance [6]. Cellulite primarily impacts women, with research indicating that approximately 80% to 90% of girls who have reached puberty have cellulite to some extent. The primary cause of this gender inequality can be attributed to variations in the distribution of adipose tissue, the form of connective tissue, and hormonal influences. Estrogen promotes the storage of fat in the thighs, hips, and buttocks, areas commonly affected by cellulite [6].

Lifestyle

Lifestyle factors play a critical role in the development of MetS. A sedentary lifestyle, poor dietary habits (high intake of sugars, fats, and processed foods), smoking, and excessive alcohol consumption significantly increase the risk. Lack of physical activity contributes to obesity, insulin resistance, and hypertension, which are core components of MetS [7]. Similarly, lifestyle choices impact the development and severity of cellulite. A diet high in processed foods, sugars, and unhealthy fats can contribute to the accumulation of fat deposits and the appearance of cellulite. Physical inactivity exacerbates the condition by promoting weight gain and reducing muscle tone, making cellulite more visible. Smoking and excessive alcohol consumption also impair blood circulation and skin health, further contributing to cellulite formation [8].

Genetic Predisposition

Genetics plays a significant role in MetS, influencing factors such as fat distribution, insulin sensitivity, and lipid metabolism. A family history of type 2 diabetes, hypertension, and cardiovascular diseases increases an individual's risk. Specific genetic variants have been associated with an increased susceptibility to MetS, affecting the body's ability to manage blood sugar, fat, and energy [9]. More specifically, research has identified several single nucleotide polymorphisms (SNPs) associated with MetS, particularly in genes involved in lipid metabolism. Notably, SNPs in genes such as GNB3, PPARG, TCF7L2, APOA5, APOC3, APOE, and CETP have shown varying degrees of association with MetS. For example, minor alleles of rs9939609 (FTO) and rs7903146 (TCF7L2) are more prevalent in individuals with MetS, while the Taq-1B (CETP) minor allele is less common among MetS patients [9]. Genetic predisposition is also a significant factor in cellulite development. Individuals with a family history of cellulite are more likely to develop the condition. Genetics influence skin structure, fat distribution, and metabolic rate, all of which contribute to cellulite formation. Variations in genes related to connective tissue structure and function can affect the severity of cellulite [10].

Pathophysiology of MetS and cellulite

Pathophysiology of MetS

Insulin resistance: This is a hallmark of MetS and plays a pivotal role in its pathogenesis. Insulin resistance is defined as the diminished ability of cells to respond to insulin action, primarily in muscle, liver, and adipose tissues [11]. Under normal conditions, insulin facilitates glucose uptake into cells for energy production. However, insulin resistance impairs this process, resulting in elevated blood glucose levels [12].

The pathogenesis of insulin resistance involves multiple mechanisms. Central obesity, a key component of MetS, results in an increase of adipose tissue, particularly visceral fat. Visceral adipocytes are metabolically active and secrete a variety of adipokines and pro-inflammatory cytokines (e.g., TNF-α, IL-6) that contribute to insulin resistance [13]. Excessive fatty acids, which are produced by increased lipolysis in adipose tissue, accumulate in non-adipose tissues such as the liver and muscle. This ectopic fat deposition leads to lipotoxicity, which impairs insulin signaling pathways [14]. Mitochondrial dysfunction in muscle cells reduces the oxidation of fatty acids, contributing to the buildup of intracellular lipid intermediates that interfere with insulin signaling [15].

Chronic inflammation: Chronic low-grade inflammation is another critical factor in MetS development. Obesity, particularly through the expansion of adipose tissue, activates inflammatory pathways. Enlarged adipocytes and infiltrating macrophages in adipose tissue produce pro-inflammatory cytokines and chemokines, perpetuating a state of chronic inflammation [16]. Cytokines such as TNF-α and IL-6 impair insulin signaling by activating serine kinases that phosphorylate insulin receptor substrate-1 (IRS-1), reducing its ability to transduce insulin signals [17]. Inflammatory mediators promote endothelial dysfunction, characterized by reduced nitric oxide bioavailability and increased oxidative stress. This contributes to hypertension and atherogenesis, which are MetS components [18].

Hormonal imbalances: The imbalance of adipokines and hormones such as cortisol and sex hormones play a significant role in MetS. Individuals with MetS typically have reduced levels of adiponectin, an anti-inflammatory and insulin-sensitizing adipokine. Conversely, leptin levels are elevated, contributing to leptin resistance and impaired regulation of appetite and energy balance [19]. Chronic stress and elevated cortisol levels can lead to central obesity, insulin resistance, and dyslipidemia by promoting lipogenesis and glucose production in the liver [20]. Post-menopausal women are at increased risk for MetS due to decreased estrogen levels, which affect fat distribution and increase the risk of insulin resistance and cardiovascular disease [21].

Pathophysiology of Cellulite

Cellulite results from structural changes in the subcutaneous tissue, involving altered connective tissue, fat deposition, and microcirculatory changes [22]. The primary structural change in cellulite is the alteration of connective tissue within the dermis and subcutaneous layers. In individuals with cellulite, the fibrous septae that separate fat lobules become thickened and rigid. These septae tether the skin to underlying structures, creating tension that pulls the skin down and results in the characteristic dimpling [23]. There is an imbalance in collagen production and degradation, leading to weakened connective tissue. Decreased collagen synthesis and increased activity of matrix metalloproteinases (MMPs) contribute to the breakdown of the extracellular matrix [24].

Cellulite is associated with increased deposition of subcutaneous fat, particularly in areas influenced by hormonal factors. Adipocytes in cellulite-prone areas undergo hypertrophy (increase in size) and hyperplasia (increase in number), leading to larger fat lobules that protrude into the dermis, exacerbating the lumpy appearance [25]. Hormonal influences, particularly estrogen, promote fat storage in specific areas such as the thighs and buttocks, where cellulite is most commonly observed [26]. Impaired microcirculation contributes significantly to the pathophysiology of cellulite. In cellulite-affected areas, there is a reduction in capillary blood flow and lymphatic drainage. This leads to the accumulation of interstitial fluid and metabolic waste products, exacerbating tissue edema and fibrosis. Increased capillary permeability allows plasma proteins to leak into the interstitial space, contributing to tissue swelling and inflammation [27].

Potential links between MetS and cellulite

Emerging hypotheses suggest a potential link between the systemic alterations in MetS and the localized manifestations of cellulite. Both conditions share several pathophysiological mechanisms, including inflammation, hormonal imbalances, and changes in adipose tissue function.

Inflammation

Chronic low-grade inflammation is a common feature of both MetS and cellulite. In MetS, systemic inflammation results from adipose tissue dysfunction and macrophage infiltration. Similarly, in cellulite, local inflammation is driven by adipocyte hypertrophy and fibrosis. Elevated levels of pro-inflammatory cytokines (e.g., TNF-α, IL-6) in MetS can exacerbate connective tissue remodeling and fibrosis in cellulite, suggesting a systemic influence on local tissue changes [28,29].

Adipose Tissue Dysfunction

Adipose tissue dysfunction is central to both MetS and cellulite. In MetS, visceral adiposity contributes to insulin resistance and inflammation. In cellulite, subcutaneous fat hypertrophy and an altered extracellular matrix contribute to the characteristic skin changes. Insulin resistance in MetS can influence fat metabolism and storage, potentially affecting areas prone to cellulite. Impaired insulin signaling can lead to increased lipogenesis and reduced lipolysis, exacerbating fat accumulation in cellulite-prone regions [30,31].

Microcirculatory Changes

Microcirculatory changes are evident in both MetS and cellulite. Endothelial dysfunction and reduced capillary density are observed in MetS, leading to impaired tissue perfusion. In cellulite, similar microcirculatory impairments, such as reduced blood flow and lymphatic drainage, contribute to tissue edema and fibrosis. These changes may be influenced by the systemic endothelial dysfunction seen in MetS [32].

Genetic Factors

Genetic factors influence the susceptibility to both MetS and cellulite. Variants in genes related to adipose tissue function, inflammation, and collagen metabolism can predispose individuals to both conditions. Common genetic variants associated with obesity, insulin resistance, and inflammation may contribute to the development of both MetS and cellulite, highlighting a potential genetic link [33].

The Role of Hormones in Cellulite

Estrogen, among other hormones, has a substantial impact on the development and intensity of cellulite. It influences the allocation of adipose tissue and the well-being of connective tissues, both of which are pivotal elements in the formation of cellulite. Estrogen facilitates adipose tissue accumulation in regions such as the thighs, hips, and buttocks, which are frequently affected by cellulite. Women may undergo changes in fat distribution that worsen cellulite throughout periods of hormonal fluctuation, such as puberty, pregnancy, and menopause. Estrogen influences adipogenesis (the formation of new fat cells) and lipogenesis (the storage of fat within cells), contributing to the accumulation of subcutaneous fat that characterizes cellulite [3]. Additionally, estrogen affects the synthesis and degradation of collagen, the main structural protein in connective tissues. High levels of estrogen can lead to decreased collagen production and increased collagen breakdown, resulting in weaker connective tissue structures. This weakening allows fat cells to protrude into the dermis, creating the dimpled appearance of cellulite [34].

Chronically high insulin levels promote the storage of fat, particularly in adipose tissues, by enhancing lipogenesis and inhibiting lipolysis. Moreover, insulin resistance is associated with increased levels of inflammatory markers, contributing to chronic low-grade inflammation. This inflammation exacerbates the remodeling of connective tissues and increases the deposition of subcutaneous fat, both of which are critical factors in the formation and severity of cellulite. Consequently, individuals with insulin resistance often experience a worsening of cellulite due to these metabolic and inflammatory disruptions [35].

Catecholamines, including adrenaline (epinephrine) and noradrenaline (norepinephrine), play a critical role in regulating blood flow and fat metabolism. These hormones are released in response to stress and physical activity, initiating a cascade of metabolic processes that include lipolysis, and the breakdown of fat stores to release fatty acids for energy. However, imbalances in catecholamine levels can significantly impact the distribution and appearance of adipose tissue. When catecholamine levels are inadequate or when there is a dysfunction in adrenergic receptors, the blood flow to adipose tissue can be reduced. This impaired blood circulation affects the delivery of oxygen and nutrients to the fat cells and hinders the removal of metabolic waste products. Consequently, the reduced blood flow contributes to the stasis and fibrosis seen in cellulite. The local decrease in catecholamine-induced lipolysis can also exacerbate fat accumulation in cellulite-prone areas. Insufficient adrenaline and noradrenaline activity means that fat cells do not adequately break down stored lipids, leading to increased fat storage. This excess fat further distends the subcutaneous tissue, creating the dimpled appearance characteristic of cellulite. Furthermore, the stagnant blood flow promotes the accumulation of interstitial fluid, leading to edema and further emphasizing the lumpy texture of the skin [36].

Thyroid hormones, primarily thyroxine (T4) and triiodothyronine (T3), are essential regulators of the body's overall metabolism. They influence numerous physiological processes, including basal metabolic rate, protein synthesis, and the metabolism of carbohydrates and fats. Hypothyroidism is a medical disorder where the thyroid gland is not producing enough hormones, resulting in low levels of thyroid hormones. This can cause several metabolic disturbances that worsen the appearance of cellulite. A key indication of hypothyroidism is an increase in body weight, frequently caused by a decrease in metabolic rate. This weight gain usually involves an increase in fat accumulation in areas that are susceptible to cellulite, such as the thighs and buttocks [37]. Additionally, hypothyroidism can cause fluid retention due to the accumulation of glycosaminoglycans in tissues, which attract water. This fluid retention contributes to the swelling and puffiness associated with cellulite. The increased interstitial fluid volume distorts the normal architecture of the subcutaneous tissue, promoting the characteristic skin dimpling of cellulite. Furthermore, thyroid hormone deficiencies can weaken the structural integrity of connective tissue by affecting collagen synthesis and remodeling. This weakening makes it easier for fat cells to push through the connective tissue matrix, exacerbating the lumpy appearance of cellulite. The impaired connective tissue, combined with increased fat and fluid retention, creates an optimal environment for the development and worsening of cellulite [38].

Swelling and fluid retention in cellulite

Swelling and fluid retention, often resulting from lymphatic and venous insufficiency, can exacerbate the appearance of cellulite. These processes contribute to the congestion of tissues, leading to increased pressure and the characteristic dimpled appearance of cellulite. The lymphatic system functions to remove surplus fluid from tissues and transport it back into the bloodstream. When the functioning of the lymphatic system is impaired, fluid has the potential to build up in the gaps between cells, resulting in the condition of swelling known as edema. This fluid retention can increase the pressure on adipose tissues and connective structures, worsening cellulite [3]. Venous insufficiency occurs when veins have difficulty sending blood from the limbs back to the heart. This condition can lead to the pooling of blood and fluid in the lower extremities, contributing to swelling and increased pressure on the skin and subcutaneous tissues. The resulting edema can make cellulite more pronounced [38].

Linking MetS to polycystic ovary syndrome (PCOS) and cellulite

Hormonal Imbalances

Both MetS and PCOS are characterized by significant hormonal imbalances. In MetS, insulin resistance plays a central role, leading to hyperinsulinemia, which can affect other hormone levels, including sex hormones. Similarly, PCOS is marked by insulin resistance and hyperinsulinemia, which can exacerbate hyperandrogenism, a hallmark of PCOS. Hyperinsulinemia in both conditions leads to an overproduction of androgens by the ovaries, contributing to symptoms such as hirsutism, acne, and irregular menstrual cycles in PCOS and potentially influencing the development of cellulite through effects on fat distribution and connective tissue health [39].

Insulin Resistance

Insulin resistance is a key component in the pathophysiology of both MetS and PCOS. Insulin resistance leads to elevated blood glucose levels and compensatory hyperinsulinemia. In PCOS, this condition exacerbates ovarian androgen production and disrupts normal follicular development, leading to anovulation and polycystic ovaries [40]. Insulin resistance is also implicated in the pathogenesis of MetS, contributing to hypertension, dyslipidemia, and increased visceral fat deposition. The resultant fat distribution and metabolic disturbances can influence the development and severity of cellulite, particularly through mechanisms involving increased adipogenesis and inflammation [1].

Diagnosis and Management

Recognizing the interconnected nature of these conditions is crucial for effective diagnosis and management. Clinicians should be aware that women presenting with signs of PCOS, such as menstrual irregularities and hyperandrogenism, are also at increased risk for MetS and vice versa. Comprehensive metabolic screening, including glucose tolerance tests, lipid profiles, and blood pressure measurements, should be part of the evaluation for women with PCOS [41]. Similarly, women diagnosed with MetS should be evaluated for symptoms of PCOS.

Implications for Treatment

The overlapping pathophysiology suggests that treatment strategies targeting insulin resistance could benefit both conditions. Metformin, a medication commonly used to improve insulin sensitivity, has shown efficacy in managing both PCOS and components of MetS. Lifestyle interventions, including diet and exercise, are foundational in managing these conditions, as they improve insulin sensitivity, reduce androgen levels, and can help reduce the severity of cellulite by promoting healthy body composition and improving circulation [42].

Relationship between BMI and cellulite

Higher BMI and Increased Risk

Individuals with a higher BMI tend to exhibit a greater amount of subcutaneous fat, which significantly contributes to the appearance of cellulite. This condition is particularly noticeable in obese individuals due to the increased accumulation of fat cells. The excess fat pushes against the connective tissue beneath the skin, resulting in the characteristic dimpled or lumpy texture of cellulite. Research indicates that the higher the BMI, the more pronounced the visibility of cellulite, primarily because of the greater volume of fat deposits that distort the overlying skin [3].

Normal Weight and Cellulite

Contrary to common belief, cellulite is not exclusive to overweight or obese individuals. Those with a normal BMI can also develop cellulite, influenced by various factors beyond body fat percentage. Genetics plays a crucial role, as individuals may inherit a predisposition to cellulite from their parents. Hormonal fluctuations, particularly estrogen, can affect fat distribution and connective tissue integrity, contributing to cellulite formation. Additionally, skin structure and the thickness of the dermis and epidermis layers can impact cellulite visibility, making it possible for individuals with a normal BMI to experience this condition [43].

Underweight Individuals

Although less prevalent, cellulite can also affect underweight individuals. In such cases, the appearance of cellulite is often due to thin skin or a genetic tendency towards weaker connective tissues. The lack of sufficient subcutaneous fat might expose the underlying connective tissue structures, making any irregularities more noticeable. Moreover, even minimal fat deposits in genetically predisposed individuals can lead to the formation of cellulite [38].

Slim women with cellulite

Prevalence and Misconceptions

A prevalent fallacy regarding cellulite is that it exclusively impacts persons who are overweight or fat. Nevertheless, cellulite can impact individuals of varying body types, even those who are slender. The dimpled or lumpy appearance of cellulite is not exclusively linked to the amount of body fat a person has, but rather to the structure of the skin and underlying tissues. Studies have shown that cellulite is prevalent in up to 90% of post-pubescent women, regardless of their weight or body composition [3]. The belief that only overweight or obese individuals develop cellulite stems from the association of excess body fat with the condition. While increased body fat can exacerbate the appearance of cellulite, it is not the sole factor. Even women with low body fat percentages can have cellulite due to other underlying causes [34].

Contributing Factors

Cellulite in slim women can be attributed to various causes, such as heredity, skin structure, and hormonal impacts. Cellulite predisposition is heavily influenced by genetics. Regardless of an individual's body weight, their likelihood of developing cellulite is strongly influenced by their family history. Specific genetic variations influence skin structure, fat distribution, and the body's ability to process and store fat. These genetic factors can predispose even thin women to cellulite [38].

Some women naturally store fat in areas prone to cellulite, such as the thighs, buttocks, and hips. This fat distribution pattern is often inherited and can occur in women of any body size [38]. Genetic factors also determine the elasticity and thickness of the skin. Women with thinner or less elastic skin are more likely to show the underlying fat lobules and connective tissue that create the appearance of cellulite [44].

The structural integrity of the skin and the underlying connective tissue play a crucial role in the development of cellulite. The dermis and subcutaneous layers of the skin interact in ways that can lead to the formation of cellulite, regardless of body fat percentage. Cellulite forms when the fibrous bands of connective tissue that connect the skin to underlying muscles pull down on the skin, creating dimples. In women, these bands are arranged in a way that predisposes them to cellulite formation more so than in men, who have a different connective tissue pattern [34]. The adipocytes in the subcutaneous layer are arranged into lobules encased by connective tissue. Cellulite occurs when lobules in the skin press against the surface while connective tissue bands pull downward, resulting in the formation of dimples [38].

Cellulite development is largely influenced by hormonal shifts and imbalances. Estrogen, in particular, plays a crucial role in fat distribution and the structural changes in the skin that lead to cellulite [40]. Hormonal changes during puberty, pregnancy, and menopause can exacerbate cellulite. These periods are associated with significant changes in fat distribution and skin structure, leading to the development or worsening of cellulite [34].

While genetics, skin structure, and hormones are significant contributors, lifestyle factors can also play a role in the development of cellulite in slim women. A diet high in processed foods, sugars, and unhealthy fats can lead to the development of cellulite by promoting inflammation and poor circulation. Even slim women can have diets that predispose them to cellulite [38].

Inadequate physical activity can result in diminished muscle tone and circulation, both of which can worsen the visibility of cellulite. Regular participation in physical exercise enhances good blood circulation, hence diminishing the intensity of cellulite [28]. Ensuring adequate hydration and adhering to a proper skincare routine is vital for preserving skin suppleness and overall well-being. Dehydration and poor skin care can make cellulite more noticeable by affecting the skin's texture and resilience [34].

Cellulite in anorexia

Incidence and Reasons

It might seem counterintuitive that individuals with anorexia nervosa, a condition characterized by extreme weight loss and low body fat, would experience cellulite. However, cellulite can occur even in this population due to various underlying factors, including body composition changes and hormonal disruptions.

Anorexia nervosa often leads to significant muscle wasting. The loss of lean muscle mass can make the remaining subcutaneous fat more noticeable. Even a small amount of fat can create a dimpled appearance if the muscle tone beneath the skin is insufficient to provide support [45]. In some cases, severe caloric restriction and malnutrition can lead to abnormal fat distribution. This redistribution can cause localized fat deposits that contribute to the appearance of cellulite, even when overall body fat is low [46].

Anorexia nervosa often leads to amenorrhea (loss of menstrual periods) and a significant drop in estrogen levels. Estrogen is crucial for maintaining skin elasticity and connective tissue health. A deficiency can weaken the connective tissue structures, allowing any remaining fat to push through and create the appearance of cellulite [45]. Malnutrition and stress associated with anorexia can lead to thyroid hormone imbalances. Hypothyroidism, or low thyroid hormone levels, can cause dry skin and decreased circulation, both of which can exacerbate the appearance of cellulite [46].

Health and Cosmetic Concerns

Managing cellulite in individuals with anorexia nervosa involves unique challenges and considerations. Treatment must be approached with sensitivity to the underlying eating disorder and the overall health of the patient. The primary focus in treating anorexia nervosa is nutritional rehabilitation and weight restoration. Ensuring that the patient receives adequate nutrition is essential for improving muscle mass, hormonal balance, and overall skin health. Nutritional interventions should be closely monitored by healthcare professionals to ensure safe and effective recovery [47]. Rapid refeeding in malnourished individuals can lead to refeeding syndrome, a potentially fatal condition characterized by electrolyte imbalances. Careful management of the refeeding process is crucial to avoid complications and support healthy weight gain [48].

As part of the recovery process, incorporating gradual and supervised physical activity can help rebuild muscle mass. Strength training practices can enhance muscular tone and support the skin, potentially reducing the appearance of cellulite [23]. Proper skin care, including hydration and the use of topical treatments, can enhance skin elasticity and health. Products containing retinoids or caffeine may offer some cosmetic improvement in cellulite appearance, although these should be used with caution in individuals with sensitive or compromised skin [34].

Addressing the psychological aspects of body image is critical. Individuals recovering from anorexia may have distorted perceptions of their bodies and may be particularly sensitive to cosmetic concerns. Providing psychological support through counseling or therapy can help patients develop a healthier body image and cope with the appearance of cellulite during recovery [47].

Therapeutic implications and treatment validity for cellulite associated with MetS

Therapeutic Implications

Managing cellulite, particularly when associated with MetS, involves a multifaceted approach that addresses hormonal imbalances, lifestyle modifications, and targeted therapies. While hormonal treatments can theoretically target the underlying issues contributing to cellulite, their efficacy varies and requires more research for standardized approaches.

Hormone Replacement Therapy (HRT)

During menopause, declining estrogen levels can lead to increased fat deposition and connective tissue changes, exacerbating cellulite. Hormone replacement therapy (HRT) aims to restore estrogen levels, potentially improving skin thickness and elasticity. However, the effectiveness of HRT in reducing cellulite remains debated, with potential risks including increased chances of breast cancer and cardiovascular diseases [49]. The complex relationship between hormone levels and fat distribution necessitates cautious consideration of HRT for cellulite treatment.

Topical Treatments

Topical treatments containing retinoids (vitamin A derivatives) can improve skin elasticity and collagen production, potentially reducing the visibility of cellulite. Retinoids enhance the thickness of the dermis and improve overall skin appearance, though their effects on cellulite are generally modest [50]. Regular application is required to maintain any visible improvements.

Diet and Exercise

Improving insulin sensitivity through diet and regular physical activity is crucial. Diets focusing on low-glycemic-index foods and consistent physical exercise can mitigate hormonal imbalances that contribute to cellulite. Exercise promotes better blood flow and lymphatic drainage, which can reduce the severity of cellulite [51]. Additionally, maintaining a healthy weight and muscle tone can improve the structural integrity of the skin.

Treatment Validity

Several treatments aim to reduce swelling and fluid retention to improve the appearance of cellulite. These treatments focus on enhancing lymphatic and venous function to reduce edema and tissue congestion.

Manual Lymphatic Drainage (MLD)

This is a therapeutic massage technique specifically developed to activate the lymphatic system and facilitate the removal of surplus fluid from tissues. Studies have shown that MLD can temporarily reduce swelling and improve the appearance of cellulite, though results are often short-lived and require regular sessions for maintenance [52]. The technique involves gentle, rhythmic massaging to encourage lymph flow.

Compression Therapy

Wearing compression garments can help improve venous return and lymphatic drainage by applying consistent pressure to affected areas. Compression therapy can reduce swelling and improve skin appearance, but its effectiveness varies among individuals. Regular use and appropriate fitting are essential for optimal results [53].

Dietary Interventions

Consuming a diet abundant in anti-inflammatory foods, such as fruits, vegetables, and omega-3 fatty acids, can effectively decrease inflammation and fluid retention throughout the body. Lowering sodium consumption can additionally inhibit fluid retention, potentially enhancing the visual aspect of cellulite. Such dietary changes support systemic health, contributing to better skin conditions [54].

Hydration and Skin Care

Staying well-hydrated helps maintain skin elasticity and reduces the likelihood of fluid retention. Adequate hydration supports the lymphatic system by effectively draining excess fluids. Certain topical treatments containing ingredients like caffeine and antioxidants can temporarily improve the appearance of cellulite by promoting blood flow and reducing fluid retention in the affected areas. These treatments should be used as part of a broader skincare regimen for best results [55].

Potential local therapy of cellulite in the context of MetS

A formula for treating cellulite, especially in the context of MetS, which involves a combination of active ingredients, plant extracts, emulsifiers, solvents, and preservatives designed to improve skin appearance and reduce cellulite, is recommended. Key active ingredients would include dihydromyricetin for its antioxidant properties, caffeine to promote lipolysis and reduce water retention, hydrolyzed collagen to enhance skin elasticity, retinoids for collagen production and skin cell turnover, coenzyme Q10 to protect against oxidative stress, and tocopheryl acetate for maintaining skin barrier function. Plant extracts such as *Theobroma cacao*, *Ilex paraguariensis*, *Mangifera indica*, *Vitis vinifera*, *Zingiber officinale*, and *Rosa canina *provide additional antioxidant and anti-inflammatory benefits. Emulsifiers and solvents such as caprylyl methicone, propylene glycol, butylene glycol, glycerin, and PEG-12 dimethicone/PPG-20 crosspolymer ensure the formula's stability and enhance ingredient penetration. Preservatives including disodium edetic acid (EDTA), caprylyl/capric triglyceride, phenoxyethanol, sodium benzoate, and potassium sorbate prevent microbial growth and prolong shelf life. Additional ingredients like denatured alcohol as a solvent, triethanolamine for pH adjustment, and carbomer for thickening provide the desired consistency and functionality. This formulation would reduce cellulite, improve skin firmness, and promote better fat metabolism, making it a comprehensive approach to managing cellulite associated with MetS [43,56-67].

Research gaps and future directions

Current Research Gaps

More research is needed to elucidate the biological pathways through which metabolic dysfunction contributes to the development and exacerbation of cellulite. Existing studies have primarily focused on the overall presence of MetS rather than investigating the individual components (e.g., insulin resistance, dyslipidemia, hypertension) and their respective roles in cellulite formation and progression. Further research is warranted to explore the specific contributions of each metabolic component to cellulite pathophysiology.

Research should encompass diverse demographic groups to assess potential variations in the relationship between MetS and cellulite across different populations. Although lifestyle factors such as diet, physical activity, and stress have been implicated in both MetS and cellulite, their interplay and specific effects on cellulite development remain poorly understood. Investigating the influence of lifestyle interventions on cellulite severity among individuals with MetS could provide valuable insights into potential therapeutic approaches.

Future Research Directions

In the exploration of the progression of cellulite in individuals with MetS, longitudinal studies are paramount. These studies should monitor participants from the initial stages of metabolic dysfunction through to the development and worsening of cellulite. By doing so, researchers can illuminate temporal relationships and identify predictive factors for the onset and severity of cellulite.

Biomarker Identification

This approach involves exploring potential biomarkers associated with both MetS and cellulite to enable early detection, risk stratification, and monitoring of disease progression. Identifying specific molecular markers will also aid in understanding the underlying pathophysiological mechanisms and pinpointing novel therapeutic targets.

Intervention Trials

Designing and implementing intervention trials is essential to evaluate the effectiveness of lifestyle modifications, pharmacological agents, or other therapeutic interventions in reducing cellulite severity among individuals with MetS. Comparative studies that assess the impact of different interventions on both metabolic parameters and cellulite outcomes will provide insights for personalized treatment approaches.

Mechanistic Studies

Conducting mechanistic studies using animal models or in vitro systems will help investigate the cellular and molecular pathways through which metabolic dysfunction leads to alterations in adipose tissue structure and function, culminating in cellulite formation. These studies are crucial for gaining mechanistic insights and identifying potential targets for therapeutic intervention.

Impact of Comorbidities

Investigating the influence of comorbidities commonly associated with MetS, such as obesity, diabetes, and cardiovascular disease, on the development and progression of cellulite is vital. Understanding how these additional health conditions interact with metabolic dysfunction to affect cellulite pathophysiology will inform comprehensive management strategies. Addressing these research gaps and following these future avenues would help us better understand the complex interaction between MetS and cellulite. This will eventually lead to more effective prevention, diagnostic, and treatment techniques for both illnesses.

## Conclusions

Understanding the link between MetS and cellulite is crucial due to shared risk factors and pathophysiological mechanisms. Longitudinal studies, biomarker identification, intervention trials, and mechanistic studies are essential for elucidating this relationship. Investigating the impact of comorbidities will further inform comprehensive management strategies. Addressing these research gaps will enhance prevention, diagnosis, and treatment approaches, improving outcomes for individuals with both conditions.
